# Supplementary material for: Using the wax moth larva Galleria mellonella infection model to detect emerging bacterial pathogens
Source: PeerJ. 2019 Jan 4;6:e6150. doi: 10.7717/peerj.6150 (PMC6322482; doi:10.7717/peerj.6150)
Supplement: Supplemental Information 1 [file peerj-07-6150-s001.docx]

**Supplemental Online Material** **Hernandez *et al.* Detecting emerging bacterial pathogens**

**Supplemental Methods**

Correlation of virulence with measures of bacterial density

Bacterial density in water and sediment wash samples was quantified in four ways: 1) total density by flow cytometry, 2) total colony counts on LB agar, 3) total colony counts on coliform agar (dark-blue, pink and white/transparent colonies that include gram-negative, non-enteric bacteria) and 4) *E. coli* and coliform colony counts (only dark-blue and pink colonies on coliform agar) (Figure S3). Generalized linear mixed effects models (glmer function from the ‘glmm’ R package) were used to test the additive effects of bacterial cell density (log_10_ + 1) and sample type (sediment versus water) on *Galleria* mortality with a binomial error structure and random intercepts fitted for sampling date, and location nested within sampling date. Separate models were fitted for each of the four different measures of bacterial cell density. Crucially, we found that *Galleria* mortality was strongly, and positively, dependent on cell density for all different density measures (Figure S3 and Table S1). We also found that *Galleria* mortality was significantly lower (for a given bacterial count) when larvae were injected with water samples compared to sediment samples. This could point at the sediment being inhabited by different, more pathogenic bacteria. The exception to this was formed by estimates based on flow cytometry (Table S1), potentially because culture-based approaches reflect total bacterial density more reliably. There was a significant relationship between *Galleria* mortality and coliform agar counts (Figure S3; Table S1) with two notable exceptions: Castle Beach water and Penryn sediment (both sampled 6 July) which presumably had high mortality due to non-coliform bacteria.

**Supplemental Results**

Isolation of pathogenic clones

Individual pathogenic clones were isolated from four samples showing high mortality, genomes of which are described in the main text. The Penryn Harbour sediment (sampled 6 July) yielded a white coloured clone on coliform agar and a ‘bubble-wrap’ colony morphology on LB agar which was identified as *Proteus* *mirabilis*. Interestingly, this clone did not cause the usual melanisation in *Galleria* but instead, the larvae turned brown, indicating a different pathogenicity mechanism. Bacterial pathogens have developed numerous ways of suppressing the insect immune system, including interference with the prophenoloxidase (PO) cascade in order to inhibit melanisation. It could be that the brown colouration is due to this clone effecting the PO cascade (Vallet-Gely etal. 2008), although this remains to be determined. Castle Beach sediment (sampled 21 June) yielded a transparent clone on LB agar which was identified as *Vibrio injenensis*. Castle Beach water (sampled 6 July) yielded a bright green clone on LB agar identified as *Pseudomonas* *aeruginosa* ST-667. Mylor Harbour sediment (sampled 6 July) yielded a dark-blue clone on coliform agar, which was identified as *Escherichia coli* ST-3304.

Vallet-Gely, I., Lemaitre, B., & Boccard, F. (2008). Bacterial strategies to overcome insect defences. *Nature Reviews Microbiology*, 6(4), 302.
