# Supplementary material for: Using the wax moth larva Galleria mellonella infection model to detect emerging bacterial pathogens
Source: PeerJ. 2019 Jan 4;6:e6150. doi: 10.7717/peerj.6150 (PMC6322482; doi:10.7717/peerj.6150)
Supplement: Supplemental Information 2 — Results of the generalized linear mixed effects models (glmer) testing the additive effects of bacterial cell density (log10 + 1) and sample type (sediment versus water) on Galleria mortality with a binomial error structure and random intercepts fitted for sampling date, and location nested within sampling data. Separate models were fitted for different estimates of bacterial density (1–4). The most parsimonious model was arrived at by sequentially deleting terms and comparing model fits using c2-tests of likelihood ratios. § The ‘Sample’ effect in the fitted models provides a measure of the extent to which water and sediment differ in their effect on Galleria mortality, where negative values indicate a lower detrimental effect of water on Galleria mortality (i.e., smaller estimated intercept) compared to sediment (Intercept). P < 0.001***, P < 0.01**. [file peerj-07-6150-s002.docx]

| **1. Flow cytometry** | Parameter estimate ± SE^§^ | *χ^2^*-test |
| --- | --- | --- |
| Intercept | -4.02 ± 1.01^***^, z = -3.99 |  |
| Density | 0.63 ± 0.23^**^, *z* = 2.78 | ***χ^2^_1,3_* = 25.34, *p* <0.001** |
| Sample | -0.01 ± 0.34^NS^, z= -0.04 | *χ^2^_1,4_*= 0.001, *p* = 0.97 |
|  |  |  |
| Date | Standard deviation <0.001 |  |
| Date/Location | Standard deviation = 0.61 |  |
| **2. LB agar counts** |  |  |
| Intercept | -3.00 ± 0.49^***^, z = -6.11 |  |
| Density | 0.47 ± 0.12^***^, *z* = 3.85 | ***χ^2^_1,4_*  = 14.48, *p* < 0.001** |
| Sample | -0.62 ± 0.20^**^, z= -3.04 | ***χ^2^_1,4_* = 9.34, *p* < 0.01** |
|  |  |  |
| Date | Standard deviation <0.001 |  |
| Date/Location | Standard deviation = 0.62 |  |
| **3. Total coliform counts** |  |  |
| Intercept | -1.97 ± 0.17^***^, z = -11.73 |  |
| Density | 0.78 ± 0.09^***^, *z* = 8.94 | ***χ^2^_1,4_*  = 48.53, *p* < 0.001** |
| Sample | -0.92 ± 0.20^***^, z= -4.62 | ***χ^2^_1,4_* = 43.39, *p* < 0.001** |
|  |  |  |
| Date | Standard deviation <0.001 |  |
| Date/Location | Standard deviation < 0.001 |  |
| **4. *E. coli* counts** |  |  |
| Intercept | -1.76 ± 0.23^***^, z = -7.76 |  |
| Density | 0.62 ± 0.09^***^, *z* = 3.82 | ***χ^2^_1,4_*  = 11.32, *p* < 0.001** |
| Sample | -0.57 ± 0.20^**^, z= -2.82 | ***χ^2^_1,4_* = 7.99, *p* < 0.01** |
|  |  |  |
| Date | Standard deviation <0.001 |  |
| Date/Location | Standard deviation = 0.52 |  |
