# Supplementary material for: Using the wax moth larva Galleria mellonella infection model to detect emerging bacterial pathogens
Source: PeerJ. 2019 Jan 4;6:e6150. doi: 10.7717/peerj.6150 (PMC6322482; doi:10.7717/peerj.6150)
Supplement: Supplemental Information 4 — Hits tabulated in white are >90% nucleotide similarity (>80% coverage) and hits tabulated in grey are >75% nucleotide similarity (>80% coverage). [file peerj-07-6150-s004.docx]

| **gene name** | **nt identity (%)** | **coverage (%)** | **acc. nr.** | **description** |
| --- | --- | --- | --- | --- |
| *sul1* | 100 | 100 | JF969163:1054-1894 | a sulfonamide resistant dihydropteroate synthase of Gram-negative bacteria linked to other resistance genes of class 1 integrons. |
| *tet(J)* | 98.16 | 100 | AF038993:1-1198 | a tetracycline efflux protein expressed in Gram-negative bacteria (*Escherichia, Morganella and Proteus*). |
| *CRP* | 80.41 | 99.84 | AP009048:4153664-4154297 | a global regulator that represses MdtEF multidrug efflux pump expression. |
| *dfrA15* | 84.63 | 100 | KF534911:40-514 | an integron-encoded dihydrofolate reductase found in *Vibrio cholerae* |
| *plasmid-encoded_cat_(pp-cat)* | 79.33 | 94.4 | D16171:383-1043 | a plasmid-encoded variant of the cat gene found in *Photobacterium damselae subsp. piscicida* |
