# Supplementary material for: Using the wax moth larva Galleria mellonella infection model to detect emerging bacterial pathogens
Source: PeerJ. 2019 Jan 4;6:e6150. doi: 10.7717/peerj.6150 (PMC6322482; doi:10.7717/peerj.6150)
Supplement: Supplemental Information 5 — Hits tabulated in white are >90% nucleotide similarity (>80% coverage) and hits tabulated in grey are >75% nucleotide similarity (>80% coverage). * = The rtxA gene which is the exotoxin is present at 88% similarity but in two parts (coverage 74.62% and 24.52%) suggesting it might be inactive. The Castle Beach clone carries a zonula occludens toxin (Zot) known to increase mammalian gut permeability (74% amino acid similarity to the fish pathogen V. anguillarum (Castillo et al., 2017) which is not present in the V. injenensis Type Strain. [file peerj-07-6150-s005.docx]

| *rtxC** | 96.97 | 100 | NP_231093 | RTX toxin activating protein |
| --- | --- | --- | --- | --- |
| *hcp-2* | 83.65 | 99.81 | NP_232418 | type VI secretion system substrate |
| *luxS* | 75.94 | 82.47 | NP_230208 | S-ribosylhomocysteinase |
| *rtxB* | 82.17 | 99.49 | NP_231091 | RTX toxin transporter |
| *rtxD* | 79.69 | 99.93 | NP_231090 | RTX toxin transporter |
| *vipA/mglA* | 75 | 99.8 | NP_232508 | type VI secretion system tubule-forming protein |
| *vipB/mglB* | 79.53 | 96.75 | NP_232509 | type VI secretion system tubule-forming protein |
| *vscN2* | 77.37 | 99.92 | NP_800848 | type III secretion system ATPase |
