# Supplementary material for: Using the wax moth larva Galleria mellonella infection model to detect emerging bacterial pathogens
Source: PeerJ. 2019 Jan 4;6:e6150. doi: 10.7717/peerj.6150 (PMC6322482; doi:10.7717/peerj.6150)
Supplement: Supplemental Information 6 — Hits tabulated in white are >90% nucleotide similarity (>80% coverage) and hits tabulated in grey are >75% nucleotide similarity (>80% coverage). [file peerj-07-6150-s006.docx]

| **gene name** | **nt identity (%)** | **coverage (%)** | **acc. nr.** | **description** |
| --- | --- | --- | --- | --- |
| *tet34* | 75.05 | 100 | AB061440:306-771 | causes the activation of Mg2+-dependent purine nucleotide synthesis which protects the protein synthesis pathway. |
| *CRP* | 79.84 | 99.53 | AP009048:4153664-4154297 | a global regulator that represses MdtEF multidrug efflux pump expression. |
