# Supplementary material for: Using the wax moth larva Galleria mellonella infection model to detect emerging bacterial pathogens
Source: PeerJ. 2019 Jan 4;6:e6150. doi: 10.7717/peerj.6150 (PMC6322482; doi:10.7717/peerj.6150)
Supplement: Supplemental Information 7 — Hits tabulated in white are >90% nucleotide similarity (>80% coverage) and hits tabulated in grey are >75% nucleotide similarity (>80% coverage). [file peerj-07-6150-s007.docx]

| **gene name** | **nt identity (%)** | **coverage (%)** | **acc. nr.** | **description** |
| --- | --- | --- | --- | --- |
| *alg44* | 99.66 | 100 | NP_252232 | alginate biosynthesis protein *Alg8* |
| *alg8* | 99.8 | 100 | NP_252231 | alginate-c5-mannuronan-epimerase *AlgG* |
| *algA* | 98.82 | 100 | NP_252241 | phosphomannose isomerase / guanosine 5'-diphospho-D-mannose pyrophosphorylase |
| *algB* | 99.19 | 100 | NP_254170 | two-component response regulator |
| *algC* | 99.14 | 100 | NP_254009 | phosphomannomutase |
| *algD* | 99.24 | 100 | NP_252230 | GDP-mannose 6-dehydrogenase |
| *algE* | 99.66 | 100 | NP_252234 | alginate biosynthetic protein AlgK precursor |
| *algF* | 99.69 | 100 | NP_252240 | alginate o-acetyltransferase |
| *algG* | 99.63 | 100 | NP_252235 | outer membrane protein AlgE |
| *algI* | 99.49 | 100 | NP_252238 | alginate o-acetyltransferase |
| *algJ* | 99.15 | 100 | NP_252239 | alginate o-acetyltransferase |
| *algK* | 99.72 | 100 | NP_252233 | alginate biosynthesis protein Alg44 |
| *algL* | 99.28 | 100 | NP_252237 | poly(beta-d-mannuronate) lyase precursor |
| *algP/algR3* | 97.17 | 98.87 | NP_253940 | alginate regulatory protein |
| *algQ* | 99.17 | 100 | NP_253942 | alginate regulatory protein |
| *algR* | 99.6 | 100 | NP_253948 | alginate biosynthesis regulatory protein AlgR |
| *algU* | 99.14 | 100 | NP_249453 | alginate biosynthesis protein AlgZ/FimS |
| *algW* | 98.72 | 100 | NP_253136 | AlgW protein [Alginate regulation] |
| *algX* | 99.16 | 100 | NP_252236 | alginate biosynthesis protein |
| *algZ* | 99.35 | 100 | NP_253949 | sigma factor AlgU [Alginate (VF0091)] [Pseudomonas aeruginosa PAO1] |
| *aprA* | 99.24 | 100 | NP_249940 | alkaline metalloproteinase precursor |
| *chpA* | 98.66 | 99.92 | NP_249104 | still frameshift probable component of chemotactic signal transduction system |
| *chpB* | 98.64 | 100 | NP_249105 | probable methylesterase |
| *chpC* | 98.82 | 100 | NP_249106 | probable chemotaxis protein |
| *chpD* | 99.12 | 100 | NP_249107 | probable transcriptional regulator |
| *chpE* | 98.86 | 100 | NP_249108 | probable chemotaxis protein |
| *clpV1* | 98.93 | 100 | NP_248780 | type VI secretion system AAA+ family ATPase |
| *dotU1* | 99.33 | 100 | NP_248768 | type VI secretion system protein |
| *exoT* | 99.42 | 100 | NP_248734 | type III secretion system effector |
| *exoU* | 99.42 | 100 | AAC16023 | type III secretion system effector |
| *exsA* | 98.69 | 100 | NP_250404 | type III secretion system regulatory protein |
| *exsB* | 98.07 | 100 | NP_250403 | type III secretion system piolitin |
| *exsC* | 97.49 | 100 | NP_250401 | type III secretion system regulatory protein |
| *exsD* | 99.16 | 100 | NP_250405 | type III secretion system regulatory protein |
| *exsE* | 96.75 | 100 | NP_250402 | type III secretion system regulatory protein |
| *fha1* | 96.19 | 96.99 | NP_248771 | type VI secretion system forkhead-associated protein |
| *fimT* | 97.06 | 100 | NP_253239 | type 4 fimbrial biogenesis protein |
| *fimU* | 99.61 | 100 | NP_253240 | type 4 fimbrial biogenesis protein |
| *fimV* | 98.27 | 100 | NP_251805 | putative Type IV pili related protein |
| *fleI/flag* | 99.73 | 100 | NP_249784 | flagellar protein |
| *fleN* | 99.29 | 100 | NP_250145 | flagellar synthesis regulator FleN |
| *fleP* | 99.33 | 100 | NP_249787 | flagellar protein FliT [Deoxyhexose linking sugar 209 Da capping structure (AI138)] |
| *fleQ* | 99.19 | 100 | NP_249788 | transcriptional regulator |
| *fleR* | 98.94 | 100 | NP_249790 | two-component response regulator |
| *fleS* | 99.09 | 100 | NP_249789 | two-component sensor |
| *flgA* | 99.28 | 100 | NP_252040 | flagellar basal body P-ring biosynthesis protein |
| *flgB* | 99.75 | 100 | NP_249768 | flagellar basal body rod protein |
| *flgC* | 100 | 100 | NP_249769 | flagellar basal-body rod protein |
| *flgD* | 99.16 | 100 | NP_249770 | flagellar basal-body rod modification protein |
| *flgE* | 98.56 | 100 | NP_249771 | flagellar hook protein |
| *flgF* | 99.07 | 100 | NP_249772 | flagellar basal-body rod protein |
| *flgG* | 99.24 | 100 | NP_249773 | flagellar basal-body rod protein |
| *flgH* | 98.85 | 100 | NP_249774 | flagellar L-ring protein precursor |
| *flgI* | 99.46 | 100 | NP_249775 | flagellar P-ring protein precursor |
| *flgJ* | 99.25 | 100 | NP_249776 | flagellar rod assembly protein/muramidase |
| *flgK* | 99.12 | 100 | NP_249777 | flagellar hook-associated protein 1 |
| *flgL* | 99.32 | 100 | NP_249778 | flagellar hook-associated protein 3 |
| *flgM* | 98.46 | 100 | NP_252041 | negative regulator of flagellin synthesis |
| *flgN* | 98.73 | 100 | NP_252042 | flagella synthesis protein |
| *flhA* | 99.01 | 100 | NP_250143 | flagellar biosynthesis protein |
| *flhB* | 98.94 | 100 | NP_250140 | flagellar biosynthetic protein |
| *flhF* | 98.92 | 100 | NP_250144 | flagellar biosynthesis protein |
| *fliA* | 99.46 | 100 | NP_250146 | flagellar biosynthesis sigma factor |
| *fliC* | 99.52 | 100 | NP_249783 | B-type flagellin |
| *fliD* | 98.95 | 100 | NP_249785 | flagellar capping protein |
| *fliE* | 99.7 | 100 | NP_249791 | flagellar hook-basal body complex protein |
| *fliF* | 99.56 | 100 | NP_249792 | flagellar M-ring protein |
| *fliG* | 99.7 | 100 | NP_249793 | flagellar motor switch protein G |
| *fliH* | 98.39 | 100 | NP_249794 | flagellar assembly protein H |
| *fliI* | 99.26 | 100 | NP_249795 | flagellum-specific ATP synthase |
| *fliJ* | 99.78 | 100 | NP_249796 | flagellar protein |
| *fliK* | 97.2 | 100 | NP_250132 | flagellar hook-length control protein |
| *fliL* | 99.62 | 100 | NP_250133 | flagellar basal body protein |
| *fliM* | 99.9 | 100 | NP_250134 | flagellar motor switch protein |
| *fliN* | 99.58 | 100 | NP_250135 | flagellar motor switch protein |
| *fliO* | 99.56 | 100 | NP_250136 | flagellar protein FliO |
| *fliP* | 99.09 | 100 | NP_250137 | flagellar biosynthetic protein |
| *fliQ* | 99.63 | 100 | NP_250138 | flagellar biosynthetic protein |
| *fliR* | 98.58 | 99.36 | NP_250139 | flagellar biosynthetic protein |
| *fliS* | 99.47 | 100 | NP_249786 | flagellar protein |
| *fptA* | 98.84 | 100 | NP_252911 | Fe(III)-pyochelin receptor precursor |
| *hcp1* | 100 | 100 | NP_248775 | type VI secretion system substrate |
| *hsiA1* | 98.65 | 100 | NP_248772 | type VI secretion system hcp secretion island protein |
| *hsiB1/vipA* | 100 | 100 | NP_248773 | type VI secretion system tubule-forming protein |
| *hsiC1/vipB* | 99.6 | 100 | NP_248774 | type VI secretion system tubule-forming protein |
| *hsiE1* | 99.05 | 100 | NP_248776 | type VI secretion system hcp secretion island protein interacting with HsiB1 to form a novel subcomplex of the T6SS |
| *hsiF1* | 99.22 | 100 | NP_248777 | type VI secretion system hcp secretion island protein, a gp25-like protein but not exhibit lysozyme activity |
| *hsiG1* | 99.03 | 100 | NP_248778 | type VI secretion system hcp secretion island protein |
| *hsiH1* | 98.57 | 100 | NP_248779 | type VI secretion system hcp secretion island protein |
| *hsiJ1* | 99.33 | 100 | NP_248769 | type VI secretion system hcp secretion island protein |
| *icmF1/tssM1* | 98.91 | 100 | NP_248767 | type VI secretion system protein |
| *lasA* | 97.85 | 100 | NP_250562 | LasA protease precursor |
| *lasB* | 98.4 | 100 | NP_252413 | elastase |
| *lasI* | 99.17 | 100 | NP_250123 | autoinducer synthesis protein |
| *lip1* | 99.57 | 100 | NP_248770 | lipoprotein |
| *mbtH-like* | 100 | 100 | NP_251102 | MbtH-like protein from the pyoverdine cluster |
| *motA* | 97.89 | 100 | NP_253641 | flagellar motor protein [Deoxyhexose linking sugar 209 Da capping structure (AI138)] |
| *motB* | 99.14 | 100 | NP_253640 | flagellar motor protein [Deoxyhexose linking sugar 209 Da capping structure (AI138)] |
| *motC* | 98.65 | 100 | NP_250151 | flagellar motor protein [Deoxyhexose linking sugar 209 Da capping structure (AI138)] |
| *motD* | 98.43 | 100 | NP_250152 | flagellar motor protein [Deoxyhexose linking sugar 209 Da capping structure (AI138)] |
| *motY* | 99.59 | 100 | NP_252216 | probable outer membrane protein precursor [Deoxyhexose linking sugar 209 Da capping structure (AI138) |
| *mucA* | 99.14 | 100 | NP_249454 | alkaline metalloproteinase precursor [Alginate (VF0091) |
| *mucB* | 98.95 | 100 | NP_249455 | anti-sigma factor MucA inhibitor of alg gene expression |
| *mucC* | 99.78 | 100 | NP_249456 | negative regulator for alginate biosynthesis MucB [Alginate (VF0091)] |
| *mucD* | 98.88 | 100 | NP_249457 | serine protease MucD precursor [Alginate regulation (CVF523)] |
| *mucE* | 98.15 | 100 | NP_252722 | small envelope protein |
| *mucP* | 99.04 | 100 | NP_252339 | metalloprotease protease |
| *pchA* | 98.95 | 100 | NP_252921 | salicylate biosynthesis isochorismate synthase |
| *pchB* | 99.67 | 100 | NP_252920 | salicylate biosynthesis protein |
| *pchC* | 99.34 | 100 | NP_252919 | pyochelin biosynthetic protein |
| *pchD* | 98.78 | 100 | NP_252918 | pyochelin biosynthesis protein |
| *pchE* | 98.54 | 100 | NP_252916 | dihydroaeruginoic acid synthetase |
| *pchF* | 98.45 | 100 | NP_252915 | pyochelin synthetase |
| *pchG* | 99.14 | 100 | NP_252914 | pyochelin biosynthetic protein |
| *pchH* | 98.66 | 100 | NP_252913 | ABC transporter ATP-binding protein [ |
| *pchI* | 98.9 | 100 | NP_252912 | ABC transporter ATP-binding protein [Pyochelin |
| *pchR* | 98.99 | 100 | NP_252917 | transcriptional regulator |
| *pcr1* | 98.92 | 100 | NP_250390 | type III secretion system protein |
| *pcr2* | 98.92 | 100 | NP_250391 | type III secretion system protein |
| *pcr3* | 98.36 | 100 | NP_250392 | type III secretion system protein |
| *pcr4* | 98.79 | 100 | NP_250393 | type III secretion system protein |
| *pcrD* | 98.96 | 100 | NP_250394 | type III secretion system protein |
| *pcrG* | 99.66 | 100 | NP_250396 | type III secretion system cytoplasmic regulator |
| *pcrH* | 99.21 | 100 | NP_250398 | type III secretion system regulatory protein |
| *pcrR* | 99.31 | 100 | NP_250395 | type III secretion system regulatory protein |
| *pcrV* | 98.98 | 100 | NP_250397 | type III secretion system hydrophilic translocator needle tip protein |
| *phzA1* | 96.16 | 90.59 | NP_252899 | phenazine biosynthesis protein |
| *phzA1* | 99.39 | 100 | NP_252899 | phenazine biosynthesis protein |
| *phzB1* | 98.36 | 100 | NP_252900 | phenazine biosynthesis protein |
| *phzC1* | 99.18 | 100 | NP_252901 | phenazine biosynthesis protein |
| *phzD1* | 98.88 | 100 | NP_252902 | phenazine biosynthesis protein |
| *phzE1* | 99.1 | 100 | NP_252903 | phenazine biosynthesis protein |
| *phzF1* | 99.16 | 100 | NP_252904 | phenazine biosynthesis protein |
| *phzG1* | 99.53 | 100 | NP_252906 | phenazine biosynthesis protein |
| *phzH* | 98.91 | 100 | NP_248741 | phenazine-modifying enzyme |
| *phzM* | 99.4 | 100 | NP_252898 | phenazine-specific methyltransferase |
| *phzS* | 98.35 | 100 | NP_252907 | flavin dependent hydroxylase |
| *pilB* | 99 | 100 | NP_253216 | type 4 fimbrial biogenesis protein |
| *pilE* | 99.06 | 100 | NP_253246 | type 4 fimbrial biogenesis protein |
| *pilF* | 98.95 | 100 | NP_252494 | type 4 fimbrial biogenesis protein |
| *pilG* | 100 | 100 | NP_249099 | twitching motility protein |
| *pilH* | 99.18 | 100 | NP_249100 | twitching motility protein |
| *pilI* | 100 | 100 | NP_249101 | twitching motility protein |
| *pilJ* | 99.51 | 100 | NP_249102 | twitching motility protein |
| *pilK* | 99.2 | 100 | NP_249103 | methyltransferase PilK |
| *pilM* | 99.81 | 100 | NP_253731 | type IV pilus inner membrane platform protein |
| *pilN* | 99.5 | 100 | NP_253730 | type IV pilus inner membrane platform protein |
| *pilO* | 99.52 | 100 | NP_253729 | type IV pilus inner membrane platform protein |
| *pilP* | 99.24 | 100 | NP_253728 | type IV pilus biogenesis protein |
| *pilQ* | 99.07 | 100 | NP_253727 | type 4 fimbrial biogenesis protein |
| *pilR* | 99.18 | 100 | NP_253237 | two-component response regulator PilR |
| *pilS* | 99.18 | 100 | NP_253236 | two-component sensor |
| *pilT* | 99.61 | 100 | NP_249086 | twitching motility protein |
| *pilU* | 99.74 | 100 | NP_249087 | twitching motility protein |
| *pilV* | 99.1 | 100 | NP_253241 | type IV pilus biogenesis protein |
| *pilW* | 98.91 | 100 | NP_253242 | type IV fimbrial biogenesis protein |
| *pilX* | 99.32 | 100 | NP_253243 | type 4 fimbrial biogenesis protein |
| *pilY1* | 93.35 | 99.43 | NP_253244 | type 4 fimbrial biogenesis protein |
| *pilY2* | 96.26 | 100 | NP_253245 | type 4 fimbrial biogenesis protein |
| *plcH* | 96.08 | 100 | NP_249535 | hemolytic phospholipase C precursor |
| *popB* | 99.15 | 100 | NP_250399 | type III secretion system hydrophobic translocator pore protein |
| *popD* | 97.41 | 100 | NP_250400 | type III secretion system hydrophobic translocator pore protein |
| *popN* | 99.42 | 100 | NP_250389 | type III secretion system outer membrane protein |
| *ppkA* | 99.23 | 100 | NP_248764 | serine/threonine protein kinase |
| *pppA* | 99.45 | 100 | NP_248765 | Pseudomonas protein phosphatase |
| *pscB* | 99.05 | 100 | NP_250406 | type III secretion system protein |
| *pscC* | 99.5 | 100 | NP_250407 | type III secretion system secretin |
| *pscD* | 99 | 100 | NP_250408 | type III secretion system basal body protein |
| *pscE* | 97.06 | 100 | NP_250409 | type III secretion system cochaperone |
| *pscF* | 99.22 | 100 | NP_250410 | type III secretion system needle filament protein |
| *pscG* | 99.71 | 100 | NP_250411 | type III secretion system chaperone PscG |
| *pscH* | 100 | 100 | NP_250412 | type III secretion system protein PscH |
| *pscI* | 99.11 | 100 | NP_250413 | type III secretion system inner rod protein PscI |
| *pscJ* | 99.33 | 100 | NP_250414 | type III secretion system inner MS ring protein |
| *pscK* | 98.09 | 99.04 | NP_250415 | type III secretion system protein |
| *pscL* | 98.61 | 100 | NP_250416 | type III secretion systemt protein |
| *pscN* | 99.24 | 100 | NP_250388 | type III secretion system ATPase |
| *pscO* | 99.37 | 100 | NP_250387 | type III secretion system protein |
| *pscP* | 94.64 | 96.49 | NP_250386 | type III secretion system protein |
| *pscQ* | 98.82 | 100 | NP_250385 | type III secretion system protein |
| *pscR* | 99.85 | 100 | NP_250384 | type III secretion system protein |
| *pscS* | 99.25 | 100 | NP_250383 | type III secretion system protein |
| *pscT* | 99.24 | 100 | NP_250382 | type III secretion system protein |
| *pscU* | 98.76 | 100 | NP_250381 | type III secretion system protein |
| *ptxR* | 98.08 | 99.79 | NP_250948 | transcriptional regulator |
| *pvcA* | 98.68 | 100 | NP_250944 | paerucumarin biosynthesis protein |
| *pvcB* | 96.98 | 100 | NP_250945 | paerucumarin biosynthesis protein |
| *pvcC* | 99.27 | 100 | NP_250946 | paerucumarin biosynthesis protein |
| *pvcD* | 97.99 | 99.85 | NP_250947 | paerucumarin biosynthesis protein |
| *pvdG* | 98.69 | 100 | NP_251115 | pyoverdine biosynthesis protein |
| *pvdH* | 99.15 | 100 | NP_251103 | diaminobutyrate-2-oxoglutarate aminotransferase |
| *pvdL* | 98.8 | 100 | NP_251114 | peptide synthase [pyoverdine (IA001)] |
| *pvdM* | 94.44 | 99.78 | NP_251083 | dipeptidase precursor [pyoverdine (IA001)] [Pseudomonas aeruginosa PAO1] |
| *pvdN* | 99.45 | 100 | NP_251084 | pyoverdine biosynthesis protein |
| *pvdO* | 97.78 | 100 | NP_251085 | pyoverdine biosynthesis protein |
| *pvdQ* | 99.13 | 100 | NP_251075 | 3-oxo-C12-homoserine lactone acylase |
| *pvdS* | 97.34 | 100 | NP_251116 | extracytoplasmic-function sigma-70 factor |
| *rhlA* | 99.1 | 100 | NP_252169 | rhamnosyltransferase chain A |
| *rhlB* | 99.61 | 100 | NP_252168 | rhamnosyltransferase chain B |
| *rhlC* | 97.03 | 100 | NP_249821 | rhamnosyltransferase 2 |
| *rhlI* | 100 | 100 | NP_252166 | autoinducer synthesis protein RhlL [Quorum sensing] |
| *tagF/pppB* | 98.68 | 100 | NP_248766 | Pseudomonas protein phosphatase |
| *tagQ* | 98.25 | 100 | NP_248760 | type VI secretion associated protein |
| *tagR* | 98.95 | 100 | NP_248761 | type IV secretion associated protein |
| *tagS* | 99.25 | 100 | NP_248762 | type IV secretion associated protein |
| *tagT* | 98.89 | 100 | NP_248763 | type six secretion associated protein |
| *toxA* | 99.01 | 100 | NP_249839 | exotoxin A precursor |
| *tse1* | 99.57 | 100 | NP_250535 | type VI secretion system effector Tse1 peptidoglycanhydrolase |
| *tse2* | 98.53 | 100 | NP_251392 | type VI secretion system effector Tse2 [HSI-1 (Hcp-secretion island 1) |
| *tse3* | 98.94 | 100 | NP_252174 | type VI secretion system effector Tse3 glycoside hydrolase |
| *vgrG1a* | 98.45 | 100 | NP_248781 | type VI secretion system substrate |
| *waaA* | 99.53 | 100 | NP_253675 | lipopolysaccharide core biosynthesis protein WaaP |
| *waaC* | 98.88 | 100 | NP_253698 | 3-deoxy-D-manno-octulosonic-acid (KDO) transferase |
| *waaF* | 99.61 | 100 | NP_253699 | heptosyltransferase I |
| *waaG* | 99.2 | 100 | NP_253697 | B-band O-antigen polymerase |
| *waaP* | 99.5 | 100 | NP_253696 | UDP-glucose:(heptosyl) LPS alpha 13-glucosyltransferase |
| *xcpA/pilD* | 99.65 | 99.31 | NP_253218 | type 4 prepilin peptidase PilD [Type IV pili |
| *xcpP* | 98.16 | 100 | NP_251794 | secretion protein |
| *xcpQ* | 97.62 | 100 | NP_251795 | general secretion pathway protein D [xcp secretion system (VF0084)] |
| *xcpR* | 98.01 | 100 | NP_251793 | general secretion pathway protein E [xcp secretion system (VF0084)] |
| *xcpS* | 99.02 | 100 | NP_251792 | general secretion pathway protein F [xcp secretion system (VF0084)] |
| *xcpT* | 99.11 | 100 | NP_251791 | general secretion pathway protein G [xcp secretion system (VF0084)] |
| *xcpU* | 98.65 | 100 | NP_251790 | general secretion pathway protein H [xcp secretion system (VF0084)] |
| *xcpV* | 98.97 | 99.49 | NP_251789 | general secretion pathway protein I [xcp secretion system (VF0084)] |
| *xcpW* | 98.88 | 100 | NP_251788 | general secretion pathway protein J [xcp secretion system (VF0084)] |
| *xcpX* | 96.51 | 100 | NP_251787 | general secretion pathway protein K [xcp secretion system (VF0084)] |
| *xcpY* | 98.87 | 100 | NP_251786 | general secretion pathway protein L [xcp secretion system (VF0084)] |
| *xcpZ* | 99.24 | 100 | NP_251785 | general secretion pathway protein M [xcp secretion system (VF0084)] |
| *phzB1* | 89.89 | 88.96 | NP_252900 | phenazine biosynthesis protein |
| *pilC* | 76.17 | 98.31 | NP_253217 | still frameshift type 4 fimbrial biogenesis protein |
| *pvdA* | 83.56 | 100 | NP_251076 | L-ornithine N5-oxygenase PvdA |
| *pvdF* | 82.49 | 100 | NP_251086 | pyoverdine synthetase F |
| *pvdP* | 85.04 | 99.76 | NP_251082 | tyrosinase required for pyoverdine maturation |
