# Supplementary material for: Using the wax moth larva Galleria mellonella infection model to detect emerging bacterial pathogens
Source: PeerJ. 2019 Jan 4;6:e6150. doi: 10.7717/peerj.6150 (PMC6322482; doi:10.7717/peerj.6150)
Supplement: Supplemental Information 8 — Hits tabulated in white are >90% nucleotide similarity (>80% coverage) and hits tabulated in grey are >75% nucleotide similarity (>80% coverage). [file peerj-07-6150-s008.docx]

| *APH(3')-IIb* | 98.64 | 100 | X90856:388-1195 | a chromosomal-encoded aminoglycoside phosphotransferase |
| --- | --- | --- | --- | --- |
| *ArmR* | 96.3 | 100 | NC_002516.2:4165719-4165881 | a 53-amino-acid antirepressor allosterically inhibits MexR dimer-DNA binding by occupying a hydrophobic binding cavity within the center of the MexR dimer. ArmR up-regulation and MexR-ArmR complex formation have previously been shown to upregulate MexAB-OprM. |
| *arnA* | 98.99 | 100 | NC_002516.2:3982021-3984010 | modifies lipid A with 4-amino-4-deoxy-L-arabinose (Ara4N) which allows gram-negative bacteria to resist the antimicrobial activity of cationic antimicrobial peptides and antibiotics such as polymyxin. |
| *basS* | 98.75 | 100 | JQ340365:1-1435 | Histidine protein kinase sensor Lipid A modification gene; part of a two-component system involved in polymyxin resistance that senses high extracellular Fe(2+) |
| *bcr-1* | 98.92 | 100 | CP012901.1:5979157-5980366 | Transmembrane protein which expels bicyclomycin from the cell leading to bicyclomycin resistance. Identified in *P. aeruginosa* strains responsible for outbreaks in Brazil often appearing with blaSPM-1 another bicyclomycin resistance gene |
| *catB7* | 97.66 | 100 | NC_002516:779463-780102 | chromosome-encoded variant of the cat gene |
| *fosA* | 99.51 | 100 | NC002516:1221691-1222099 | enzyme that confers resistance to fosfomycin in *Serratia marcescens* by breaking the epoxide ring of the molecule. It depends on the cofactors Manganese (II) and Potassium and uses Glutathione (GSH) as the nucleophilic molecule. In *P. aeruginosa* FosA catalyzes the conjugation of glutathione to carbon-1 of fosfomycin rendering it ineffective as an antibacterial drug. |
| *MexA* | 99.31 | 100 | NC_002516:472024-473176 | membrane fusion protein of the MexAB-OprM multidrug efflux complex |
| *MexB* | 99.01 | 100 | L11616:1570-4711 | inner membrane multidrug exporter of the efflux complex MexAB-OprM |
| *MexC* | 99.23 | 100 | U57969:295-1459 | membrane fusion protein of the MexCD-OprJ multidrug efflux complex |
| *MexD* | 97.73 | 99.94 | U57969:1486-4618 | multidrug inner membrane transporter of the MexCD-OprJ complex |
| *MexE* | 99.12 | 100 | NC_002516:2808743-2809988 | membrane fusion protein of the MexEF-OprN multidrug efflux complex |
| *MexF* | 99.28 | 100 | NC_002516:2810009-2813198 | multidrug inner membrane transporter of the MexEF-OprN complex. mexF corresponds to 2 loci in *P. aeruginosa* PAO1 (mexF/mexB) and 4 loci in *P. aeruginosa* LESB58 (mexD/mexB). |
| *mexG* | 99.78 | 100 | NC_002516:4705956-4706403 | membrane protein required for MexGHI-OpmD efflux activity |
| *mexH* | 99.1 | 100 | NC_002516:4706410-4707523 | membrane fusion protein of the efflux complex MexGHI-OpmD |
| *mexI* | 99.39 | 100 | NC_002516:4707535-4710625 | inner membrane transporter of the efflux complex MexGHI-OpmD |
| *mexJ* | 98.91 | 100 | NC_002516.2:4119270-4120374 | membrane fusion protein of the MexJK multidrug efflux protein |
| *mexK* | 98.7 | 100 | AE004091.2:4116188-4119266 | inner membrane resistance-nodulation-cell division (RND) transporter in the MexJK multidrug efflux protein |
| *mexL* | 99.53 | 100 | AE004091.2:4120469-4121108 | a specific repressor of mexJK transcription and autoregulates its own expression |
| *mexM* | 99.31 | 100 | AB219523.1:22-1180 | membrane fusion protein of the MexMN-OprM multidrug efflux complex |
| *mexN* | 99.29 | 100 | AB219523.1:1176-4287 | inner membrane transporter of the MexMN-OprM multidrug efflux complex |
| *mexP* | 98.88 | 100 | AB219524.1:23-1181 | membrane fusion protein of the MexPQ-OpmE multidrug efflux complex |
| *mexQ* | 98.89 | 100 | AB219524.1:1177-4339 | inner membrane transporter of the multidrug efflux pump MexPQ-OpmE |
| *mexV* | 98.67 | 100 | AE004091.2:4903466-4904597 | membrane fusion protein of the MexVW-OprM multidrug efflux complex |
| *mexW* | 99.48 | 100 | NC_002516.2:4904647-4907704 | RND-type membrane protein of the efflux complex MexVW-OprM |
| *mexX* | 97.71 | 100 | AB015853:146-1316 | membrane fusion protein of the MexXY-OprM multidrug efflux complex |
| *mexY* | 98.31 | 99.81 | AB015853:1331-4472 | RND-type membrane protein of the efflux complex MexXY-OprM |
| *MuxA* | 98.98 | 100 | NC_002516.2:2854011-2855292 | membrane fusion protein component of the efflux pump system MuxABC-OpmB in *P. aeruginosa* |
| *MuxB* | 99.33 | 100 | NC_002516.2:2850883-2854015 | one of the two necessary RND components in the *P. aeruginosa* efflux pump system MuxABC-OpmB. |
| *MuxC* | 98.94 | 100 | NC_002516.2:2847776-2850887 | one of the two necessary RND components of the MuxABC-OpmB efflux pumps system in *P. aeruginosa* |
| *OpmB* | 99.13 | 100 | NC_002516.2:2846283-2847780 | outer membrane efflux protein in *P. aeruginosa* that shows functional cooperation with MuxABC to form the efflux pump system MuxABC-OpmB. |
| *opmD* | 98.57 | 99.93 | NC_002516:4710621-4712085 | outer membrane channel protein of the efflux complex MexGHI-OpmD. |
| *opmE* | 99.25 | 100 | AB219524.1:4335-5811 | outer membrane factor protein that is part of the multidrug efflux pump MexPQ-OpmE. |
| *OpmH* | 99.45 | 100 | NC_002516.2:5584101-5585550 | outer membrane efflux protein required for triclosan-specific efflux pump function. |
| *OprJ* | 98.61 | 100 | U57969:4623-6063 | outer membrane channel component of the MexCD-OprJ multidrug efflux complex. |
| *OprM* | 99.18 | 100 | NC_002516:476333-477791 | outer membrane factor protein found in *P. aeruginosa* and *Burkholderia vietnamiensis*. It is part of the MexAB-OprM MexVW-OprM MexXY-OprM and the AmrAB-OprM complex. |
| *OprN* | 98.45 | 100 | NC_002516:2813194-2814613 | outer membrane channel component of the MexEF-OprN multidrug efflux complex. |
| *OXA-50* | 98.23 | 100 | AY306130:1-790 | beta-lactamase found in *P. aeruginosa*. It confers decreased susceptibility to ampicillin and ticarcillin and interestingly to moxalactam and meropenem in *P. aeruginosa* but not in *E. coli.* Also confers resistance to piperacillin-tazobactam and cephalotin. |
| *PDC-5* | 99.41 | 100 | FJ666068:1-1195 | extended-spectrum beta-lactamase found in *P. aeruginosa* |
| *PmpM* | 99.16 | 100 | NC_002516.2:1472547-1473981 | multidrug efflux pump belonging to the MATE family of *P. aeruginosa*. PmpM is an H+ drug antiporter and is the first reported case of an H+ coupled efflux pump in the MATE family. PmpM confers resistance to fluoroquinolones fradiomycin benzalkonium chloride chlorhexidine gluconate ethidium bromide tetraphenylphosphonium chloride (TPPCl) and rhodamine 6G |
| *Pseudomonas_aeruginosa_CpxR* | 99.56 | 100 | LT673656.1:1884345-1885023 | directly involved in activation of expression of RND efflux pump MexAB-OprM in *P. aeruginosa*. CpxR is required to enhance mexAB-oprM expression and drug resistance in the absence of repressor MexR. |
| *Pseudomonas_aeruginosa_emrE* | 99.1 | 100 | NC_002516.2:1-334 | a small multidrug transporter that functions as a homodimer and that couples the efflux of small polyaromatic cations from the cell with the import of protons down an electrochemical gradient. Confers resistance to tetraphenylphosphonium methyl viologen gentamicin kanamycin and neomycin. |
| *Pseudomonas_aeruginosa_soxR* | 98.94 | 100 | NC_002516.2:2503425-2503896 | SoxR is a redox-sensitive transcriptional activator that induces expression of a small regulon that includes the RND efflux pump-encoding operon mexGHI-opmD. SoxR was shown to be activated by pyocyanin. |
| *TriA* | 99.22 | 100 | NC_002516.2:177307-178459 | membrane protein that is fused to TriB and both are required for the triclosan efflux pump function of TriABC-OpmH in *P. aeruginosa* |
| *TriB* | 99.25 | 100 | NC_002516.2:178455-179526 | membrane protein that is fused to TriA and both are required for the triclosan efflux pump function of TriABC-OpmH in *P. aeruginosa* |
| *TriC* | 99.18 | 100 | NC_002516.2:179522-182570 | resistance nodulation cell division (RND) transporter that is a part of TriABC-OpmH a triclosan-specific efflux protein. |
| AxyY | 78.44 | 97.74 | AFRQ01000061.1:23987-27125 | periplasmic adaptor protein of the AxyXY-OprZ efflux pump system in *Achromobacter spp.* |
