# Supplementary material for: Using the wax moth larva Galleria mellonella infection model to detect emerging bacterial pathogens
Source: PeerJ. 2019 Jan 4;6:e6150. doi: 10.7717/peerj.6150 (PMC6322482; doi:10.7717/peerj.6150)
Supplement: Supplemental Information 9 — Hits tabulated in white are >90% nucleotide similarity (>80% coverage) and hits tabulated in grey are >75% nucleotide similarity (>80% coverage). [file peerj-07-6150-s009.docx]

| **gene name** | **nt identity (%)** | **coverage (%)** | **acc. nr.** | **description** |
| --- | --- | --- | --- | --- |
| *aslA* | 98.25 | 100 | AAG10151 | putative arylsulfatase |
| *chuA* | 99.74 | 100 | NP_756170 | Outer membrane heme/hemoglobin receptor |
| *chuS* | 99.51 | 100 | NP_756169 | heme oxygenase |
| *chuT* | 99.5 | 100 | NP_756175 | periplasmic heme-binding protein |
| *chuU* | 99.09 | 100 | NP_756179 | heme permease protein |
| *chuV* | 98.38 | 100 | NP_756180 | ATP-binding hydrophilic protein |
| *chuW* | 99.33 | 100 | NP_756176 | Putative oxygen independent coproporphyrinogen III oxidase |
| *chuX* | 100 | 100 | NP_756177 | putative heme-binding protein |
| *chuY* | 100 | 100 | NP_756178 | ChuY |
| *entA* | 98.26 | 100 | NP_752614 | 23-dihydro-23-dihydroxybenzoate dehydrogenase [Enterobactin (VF0228)] |
| *entB* | 99.77 | 100 | NP_752613 | isochorismatase [Enterobactin (VF0228)] |
| *entC* | 98.57 | 100 | NP_752611 | isochorismate synthase 1 [Enterobactin (VF0228)] |
| *entD* | 99.87 | 100 | NP_752599 | phosphopantetheinyl transferase component of enterobactin synthase multienzyme complex [Enterobactin (VF0228)] |
| *entE* | 98.39 | 100 | NP_752612 | 23-dihydroxybenzoate-AMP ligase component of enterobactin synthase multienzyme complex [Enterobactin (VF0228)] |
| *entF* | 98.7 | 99.28 | NP_752604 | enterobactin synthase multienzyme complex component ATP-dependent [Enterobactin (VF0228)] |
| *entS* | 98.32 | 100 | NP_752609 | enterobactin exporter iron-regulated [enterobactin (IA019)] |
| *fdeC* | 98.28 | 100 | YP_002390132 | adhesin |
| *fepA* | 99.06 | 100 | NP_752600 | ferrienterobactin outer membrane transporter [Enterobactin (VF0228)] |
| *fepB* | 99.48 | 100 | NP_752610 | ferrienterobactin ABC transporter periplasmic binding protein [Enterobactin (VF0228)] |
| *fepC* | 98.9 | 100 | NP_752606 | ferrienterobactin ABC transporter ATPase [Enterobactin (VF0228)] |
| *fepD* | 98.92 | 100 | NP_752608 | ferrienterobactin ABC transporter permease [Enterobactin (VF0228)] |
| *fepG* | 98.49 | 100 | NP_752607 | iron-enterobactin ABC transporter permease [Enterobactin (VF0228)] |
| *fes* | 99.58 | 100 | NP_752602 | enterobactin/ferric enterobactin esterase [Enterobactin (IA019)] |
| *fimA* | 92.08 | 100 | NP_757241 | Type-1 fimbrial protein A chain precursor |
| *fimB* | 99.83 | 100 | NP_757239 | Type 1 fimbriae Regulatory protein |
| *fimC* | 99.04 | 100 | NP_757243 | Chaperone protein fimC precursor |
| *fimD* | 99.01 | 100 | NP_757244 | Outer membrane usher protein fimD precursor |
| *fimE* | 100 | 100 | NP_757240 | Type 1 fimbriae Regulatory protein |
| *fimF* | 99.81 | 100 | NP_757245 | FimF protein precursor |
| *fimG* | 98.81 | 100 | NP_757247 | FimG protein precursor |
| *fimH* | 98.9 | 100 | NP_757248 | FimH protein precursor |
| *fimI* | 99.44 | 100 | NP_757242 | Fimbrin-like protein fimI precursor |
| *fyuA* | 99.8 | 100 | NP_405467 | pesticin/yersiniabactin receptor protein |
| *gspM* | 92.62 | 92.91 | YP_404609 | general secretion pathway protein M |
| *ibeA* | 98.47 | 99.93 | AAF98391 | invasion protein |
| *irp1* | 99.69 | 100 | NP_405471 | yersiniabactin biosynthetic protein |
| *irp2* | 99.44 | 100 | NP_405472 | yersiniabactin biosynthetic protein |
| *ompA* | 99.81 | 100 | AAF37887 | outer membrane protein A |
| *set1A* | 94.38 | 99.44 | YP_006098866 | toxin subunit |
| *set1B* | 98.39 | 100 | YP_006098865 | toxin subunit |
| *vat* | 99.9 | 100 | NP_752330 | Haemoglobin protease |
| *yagV/ecpE* | 98.14 | 99.74 | NP_286006 | *E. coli* common pilus chaperone |
| *yagW/ecpD* | 99.09 | 100 | NP_286007 | polymerized tip adhesin of ECP fibers |
| *yagX/ecpC* | 97.78 | 100 | NP_286008 | *E. coli* common pilus usher |
| *yagY/ecpB* | 97.76 | 100 | NP_286009 | *E. coli* common pilus chaperone |
| *yagZ/ecpA* | 98.3 | 100 | NP_286010 | *E. coli* common pilus structural subunit |
| *ybtA* | 99.58 | 100 | NP_405473 | transcriptional regulator |
| *ybtE* | 99.94 | 100 | NP_405468 | yersiniabactin siderophore biosynthetic protein |
| *ybtP* | 99.61 | 100 | NP_405474 | lipoprotein inner membrane ABC-transporter |
| *ybtQ* | 99.72 | 100 | NP_405475 | inner membrane ABC-transporter |
| *ybtS* | 99.69 | 100 | NP_405477 | salicylate synthase Irp9 |
| *ybtT* | 99.75 | 100 | NP_405469 | yersiniabactin biosynthetic protein |
| *ybtU* | 99.73 | 100 | NP_405470 | yersiniabactin biosynthetic protein |
| *ybtX* | 99.69 | 100 | NP_405476 | putative signal transducer |
| *ykgK/ecpR* | 99.32 | 100 | NP_286011 | regulator protein |
| *cheW* | 75.11 | 90.36 | YP_001006779 | purine-binding chemotaxis protein |
| *cheY* | 77.06 | 99.49 | YP_001006774 | chemotaxis regulatory protein |
| *csgB* | 83.59 | 99.78 | NP_460114 | minor curlin subunit precursor curli nucleator protein |
| *csgD* | 81.41 | 100 | NP_460113 | DNA-binding transcriptional regulator |
| *csgE* | 79.29 | 98.48 | NP_460112 | curli production assembly/transport protein |
| *csgF* | 81 | 99.04 | NP_460111 | curli production assembly/transport protein |
| *csgG* | 83.45 | 100 | NP_460110 | curli production assembly/transport protein |
| *flgG* | 75.81 | 98.93 | YP_001006759 | flagellar basal-body rod protein |
| *flgH* | 78.99 | 80.7 | YP_001006758 | flagellar L-ring protein precursor |
| *flhA* | 75.01 | 99.04 | YP_001006770 | flagellar biosynthesis protein |
| *flhC* | 75.05 | 95.19 | YP_001006783 | flagellar biosynthesis transcription activator |
| *fliG* | 78.54 | 99.5 | YP_001006742 | flagellar motor switch protein G |
| *fliI* | 75.74 | 92.79 | YP_001006744 | flagellum-specific ATP synthase |
| *fliM* | 76.33 | 99.5 | YP_001006748 | flagellar motor switch protein |
| *fliP* | 77.43 | 98.4 | YP_001006751 | flagellar biosynthetic protein |
