# Supplementary material for: Using the wax moth larva Galleria mellonella infection model to detect emerging bacterial pathogens
Source: PeerJ. 2019 Jan 4;6:e6150. doi: 10.7717/peerj.6150 (PMC6322482; doi:10.7717/peerj.6150)
Supplement: Supplemental Information 10 — Hits tabulated in white are >90% nucleotide similarity (>80% coverage) and hits tabulated in grey are >75% nucleotide similarity (>80% coverage). [file peerj-07-6150-s010.docx]

| **gene name** | **nt identity (%)** | **coverage (%)** | **acc. nr.** | **description** |
| --- | --- | --- | --- | --- |
| *acrB* | 98.83 | 100 | NC_000913.3:481254-484404 | Protein subunit of AcrA-AcrB-TolC multidrug efflux complex. *AcrB* functions as a herterotrimer which forms the inner membrane component and is primarily responsible for substrate recognition and energy transduction by acting as a drug/proton antiporter. |
| *acrD* | 98.56 | 100 | NC_007779:2586251-2589365 | aminoglycoside efflux pump expressed in *E. coli*. Its expression can be induced by indole and is regulated by baeRS and *cpxAR.* |
| *acrE* | 98.79 | 100 | U00096:3413864-3415022 | membrane fusion protein similar to AcrA. |
| *acrF* | 96.59 | 100 | U00096:3415033-3418138 | inner membrane transporter similar to AcrB. |
| *acrS* | 98.34 | 100 | U00096:3412803-3413466 | repressor of the AcrAB efflux complex and is associated with the expression of AcrEF. AcrS is believed to regulate a switch between AcrAB and AcrEF efflux. |
| *bacA* | 98.17 | 99.76 | U00096:3203310-3204132 | gene that recycles undecaprenyl pyrophosphate during cell wall biosynthesis which confers resistance to bacitracin |
| *baeR* | 97.23 | 99.72 | NC_007779:2166413-2167136 | response regulator that promotes the expression of MdtABC and AcrD efflux complexes. |
| *baeS* | 90.46 | 100 | AP009048:2165013-2166417 | sensor kinase in the BaeSR regulatory system. While it phosphorylates BaeR to increase its activity BaeS is not necessary for overexpressed BaeR to confer resistance. |
| *cpxA* | 98.47 | 99.56 | NC_002695:4903562-4904936 | a membrane-localized sensor kinase that is activated by envelope stress. It starts a kinase cascade that activates CpxR which promotes efflux complex expression. |
| *CRP* | 99.05 | 100 | AP009048:4153664-4154297 | global regulator that represses MdtEF multidrug efflux pump expression. |
| *emrA* | 98.13 | 100 | AP009048:2810083-2811256 | membrane fusion protein providing an efflux pathway with EmrB and TolC between the inner and outer membranes of *E. coli*. |
| *emrB* | 97.99 | 100 | U00096:2812616-2814155 | translocase in the emrB -TolC efflux protein in *E. coli*. It recognizes substrates including carbonyl cyanide m-chlorophenylhydrazone (CCCP) nalidixic acid and thioloactomycin. |
| *emrD* | 97.82 | 100 | GG749185.1:116813-118004 | multidrug transporter from the Major Facilitator Superfamily (MFS) primarily found in *E. coli*. EmrD couples efflux of amphipathic compounds with proton import across the plasma membrane. |
| *emrK* | 97.64 | 100 | D78168:537-1593 | membrane fusion protein that is a homolog of EmrA. Together with the inner membrane transporter EmrY and the outer membrane channel TolC it mediates multidrug efflux. |
| *emrR* | 99.06 | 100 | NC_000913.3:2810770-2811301 | negative regulator for the EmrAB-TolC multidrug efflux pump in *E. coli*. Mutations lead to EmrAB-TolC overexpression. |
| *emrY* | 97.66 | 100 | D78168:1592-3131 | multidrug transport that moves substrates across the inner membrane of the Gram-negative *E. coli*. It is a homolog of emrB. |
| *Escherichia_coli_acrA* | 99.08 | 100 | NC_000913.3:484426-485620 | subunit of the AcrAB-TolC multidrug efflux system that in *E. coli*. |
| *Escherichia_coli_ampC* | 98.06 | 100 | NC_000913.3:4377811-4378945 | class C ampC beta-lactamase (cephalosporinase) enzyme described in *E. coli* shown clinically to confer resistance to penicillin-like and cephalosporin-class antibiotics. |
| *Escherichia_coli_mdfA* | 96.35 | 100 | JQ394987:1-1234 | Multidrug efflux pump in *E. coli*. This multidrug efflux system was originally identified as the Cmr/CmlA chloramphenicol exporter. |
| *evgA* | 99.03 | 100 | NC_002695:3211892-3212507 | when phosphorylated is a positive regulator for efflux protein complexes emrKY and mdtEF. While usually phosphorylated in a EvgS dependent manner it can be phosphorylated in the absence of EvgS when overexpressed. |
| *evgS* | 96.19 | 100 | U00096:2484374-2487968 | sensor protein that phosphorylates the regulatory protein EvgA. evgS corresponds to 1 locus in *P. aeruginosa* PAO1 and 1 locus in *P. aeruginosa* LESB58. |
| *gadW* | 99.86 | 100 | CP015085.1:2551712-2552441 | AraC-family regulator that promotes mdtEF expression to confer multidrug resistance. GadW inhibits GadX-dependent activation. GadW clearly represses gadX and in situations where GadX is missing activates gadA and gadBC. |
| *gadX* | 93.7 | 100 | NC_007779:3974605-3975430 | AraC-family regulator that promotes mdtEF expression to confer multidrug resistance. |
| *H-NS* | 99.28 | 100 | NC_002695:1737554-1737968 | histone-like protein involved in global gene regulation in Gram-negative bacteria. It is a repressor of the membrane fusion protein genes acrE mdtE and emrK as well as nearby genes of many RND-type multidrug exporters. |
| *kdpE* | 95.84 | 99.26 | NC_000913.3:721056-721734 | transcriptional activator that is part of the two-component system KdpD/KdpE that is studied for its regulatory role in potassium transport and has been identified as an adaptive regulator involved in the virulence and intracellular survival of pathogenic bacteria. kdpE regulates a range of virulence loci through direct promoter binding. |
| *marA* | 98.18 | 100 | NC_007779:1621288-1621672 | In the presence of antibiotic stress E. coli overexpresses the global activator protein MarA which besides inducing MDR efflux pump AcrAB also down- regulates synthesis of the porin OmpF. |
| *mdtA* | 95.84 | 100 | U00096:2154016-2155264 | membrane fusion protein of the multidrug efflux complex mdtABC. |
| *mdtB* | 95.81 | 100 | U00096:2155263-2158386 | transporter that forms a heteromultimer complex with MdtC to form a multidrug transporter. MdtBC is part of the MdtABC-TolC efflux complex. |
| *mdtC* | 93.86 | 100 | U00096:2158386-2161464 | transporter that forms a heteromultimer complex with MdtB to form a multidrug transporter. MdtBC is part of the MdtABC-TolC efflux complex. In the absence of MdtB MdtC can form a homomultimer complex that results in a functioning efflux complex with a narrower drug specificity. mdtC corresponds to 3 loci in Pseudomonas aeruginosa PAO1 (gene name: muxC/muxB) and 3 loci in Pseudomonas aeruginosa LESB58. |
| *mdtE* | 98.53 | 100 | AP009048:3980026-3981184 | membrane fusion protein of the MdtEF multidrug efflux complex. It shares 70% sequence similarity with AcrA. |
| *mdtF* | 97.24 | 100 | U00096:3660414-3663528 | multidrug inner membrane transporter for the MdtEF-TolC efflux complex. |
| *mdtG* | 98.37 | 100 | NC_007779:1115841-1117068 | also named YceE, appears to be a member of the major facilitator superfamily of transporters and it has been reported when overexpressed to increase fosfomycin and deoxycholate resistances. mdtG is a member of the marA-soxS-rob regulon. |
| *mdtH* | 98.1 | 100 | U00096:1124118-1125327 | Multidrug resistance protein |
| *mdtN* | 95.83 | 100 | AP009048:4306557-4307589 | Multidrug resistance efflux pump. Could be involved in resistance to puromycin acriflavine and tetraphenylarsonium chloride. |
| *mdtO* | 97.17 | 100 | AP009048:4304506-4306558 | Multidrug resistance efflux pump. Could be involved in resistance to puromycin acriflavine and tetraphenylarsonium chloride |
| *mdtP* | 97.48 | 100 | AP009048:4303043-4304510 | Multidrug resistance efflux pump. Could be involved in resistance to puromycin acriflavine and tetraphenylarsonium chloride |
| *msbA* | 97.77 | 100 | NC_000913.3:966621-968370 | multidrug resistance transporter homolog from *E. coli* and belongs to a superfamily of transporters that contain an adenosine triphosphate (ATP) binding cassette (ABC) which is also called a nucleotide-binding domain (NBD). MsbA is a member of the MDR-ABC transporter group by sequence homology. MsbA transports lipid A a major component of the bacterial outer cell membrane and is the only bacterial ABC transporter that is essential for cell viability. |
| *patA* | 97.03 | 100 | NC_000913.3:3219494-3220874 | PatA is an ABC transporter of *Streptococcus pneumoniae* that interacts with PatB to confer fluoroquinolone resistance. |
| *PmrC* | 91.12 | 100 | AP009048:4338625-4340269 | mediates the modification of Lipid A by the addition of 4-amino-4-deoxy-L-arabinose (L-Ara4N) and phosphoethanolamine resulting in a less negative cell membrane and decreased binding of polymyxin B. |
| *pmrE* | 94.09 | 100 | U00096:2098447-2099614 | required for the synthesis and transfer of 4-amino-4-deoxy-L-arabinose (Ara4N) to Lipid A which allows gram-negative bacteria to resist the antimicrobial activity of cationic antimicrobial peptides and antibiotics such as polymyxin |
| *pmrF* | 97.63 | 100 | U00096:2367071-2368040 | required for the synthesis and transfer of 4-amino-4-deoxy-L-arabinose (Ara4N) to Lipid A which allows gram-negative bacteria to resist the antimicrobial activity of cationic antimicrobial peptides and antibiotics such as polymyxin. pmrF corresponds to 1 locus in Pseudomonas aeruginosa PAO1 and 1 locus in Pseudomonas aeruginosa LESB58. |
| *tolC* | 98.39 | 100 | FJ768952:1-1489 | TolC is a protein subunit of many multidrug efflux complexes in Gram negative bacteria. It is an outer membrane efflux protein and is constitutively open. Regulation of efflux activity is often at its periplasmic entrance by other components of the efflux complex. |
| *yojI* | 97.57 | 99.94 | NC_000913.3:1-1645 | mediates resistance to the peptide antibiotic microcin J25 when it is expressed from a multicopy vector. YojI is capable of pumping out microcin molecules. The outer membrane protein TolC in addition to YojI is required for export of microcin J25 out of the cell. Microcin J25 is thus the first known substrate for YojI. |
| *mdtK* | 75.27 | 98.74 | CP014358.1:2161326-2162751 | multidrug and toxic compound extrusions (MATE) transporter conferring resistance to norfloxacin doxorubicin and acriflavine. |
| *vgaC* | 83.55 | 100 | KU302801:102200-102431 | efflux protein expressed in staphylococci that confers resistance to streptogramin A antibiotics and related compounds. It is associated with plasmid DNA. |
