# Supplementary figures and images for: Using the wax moth larva Galleria mellonella infection model to detect emerging bacterial pathogens
Source: PeerJ. 2019 Jan 4;6:e6150. doi: 10.7717/peerj.6150 (PMC6322482; doi:10.7717/peerj.6150)

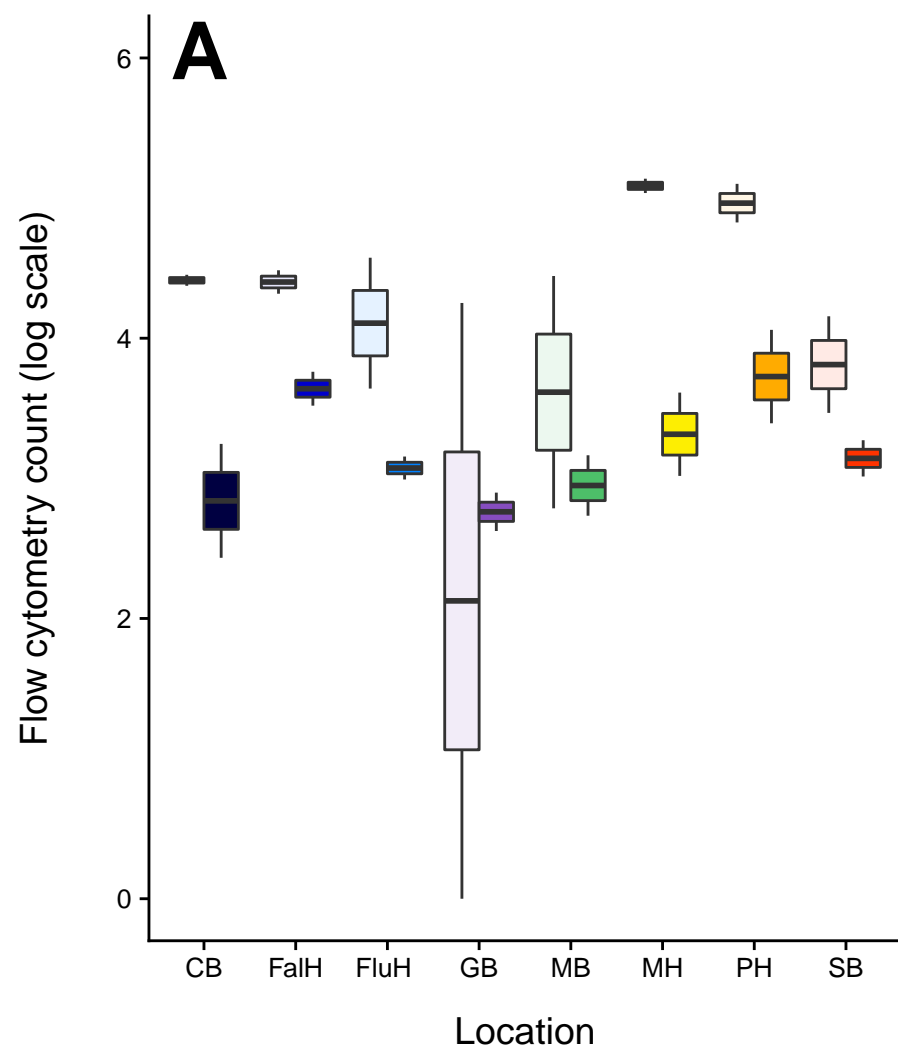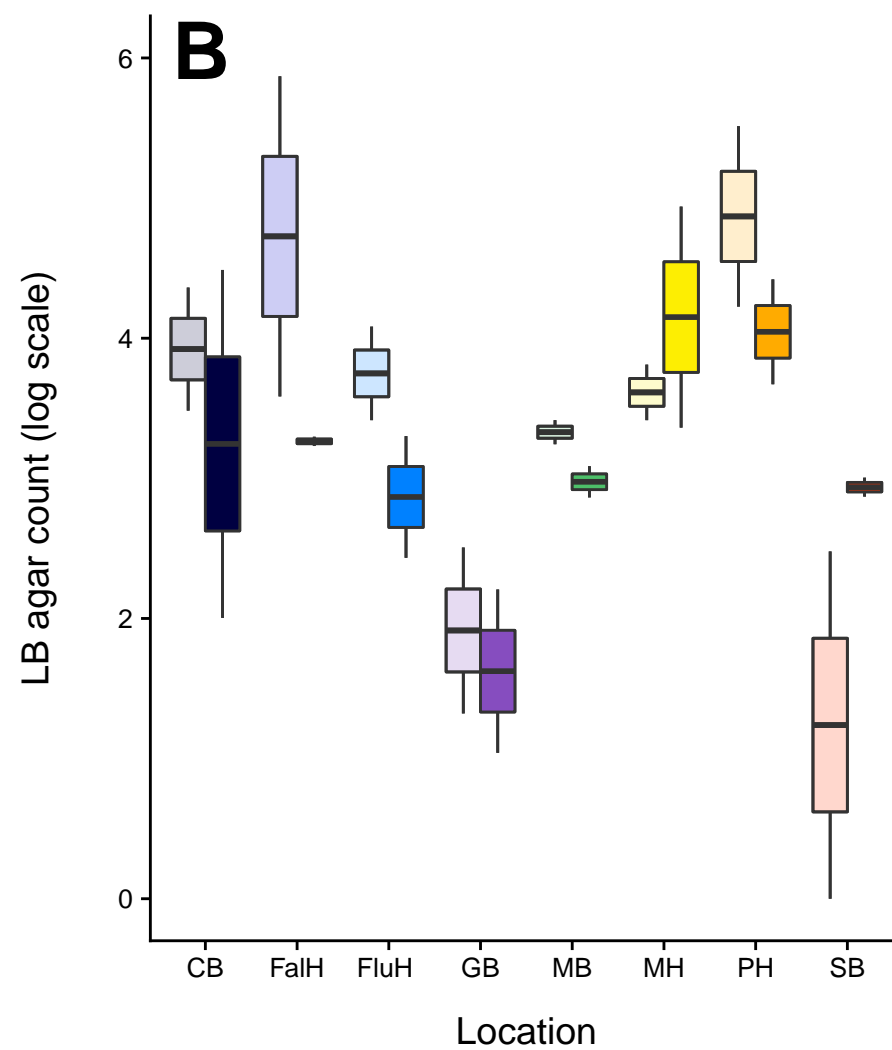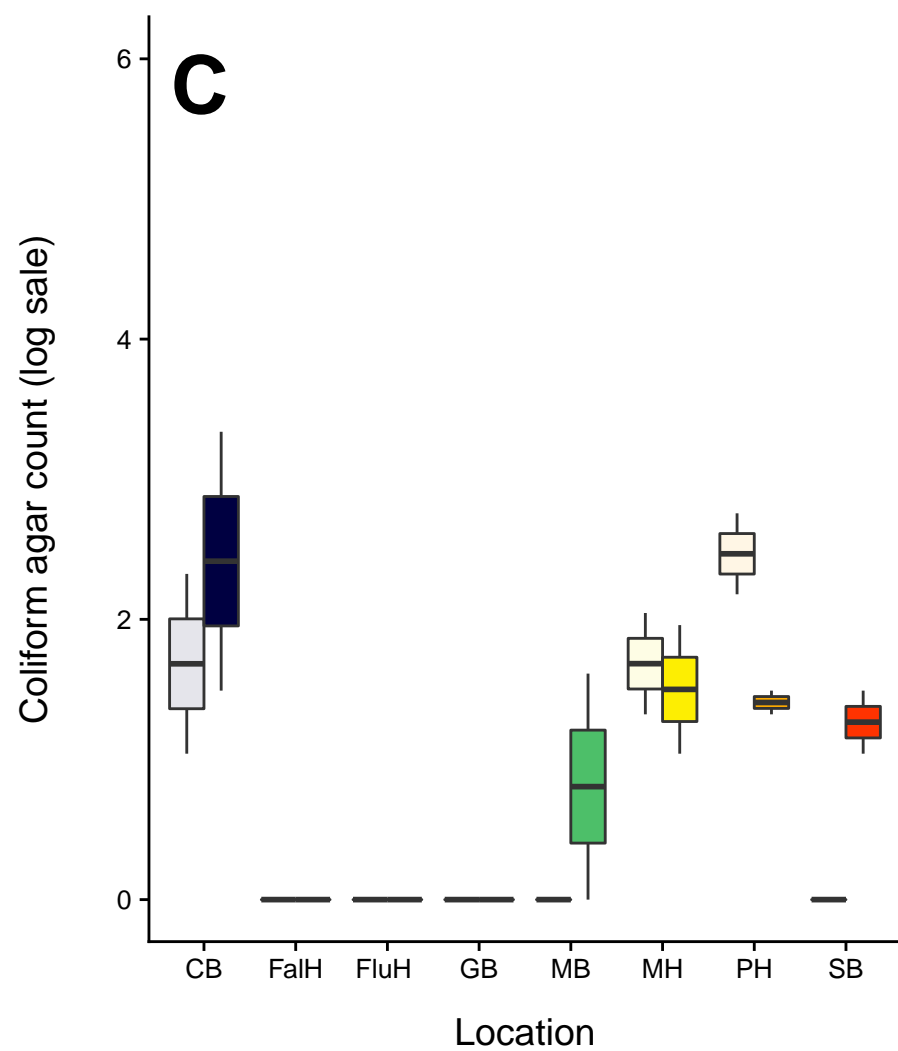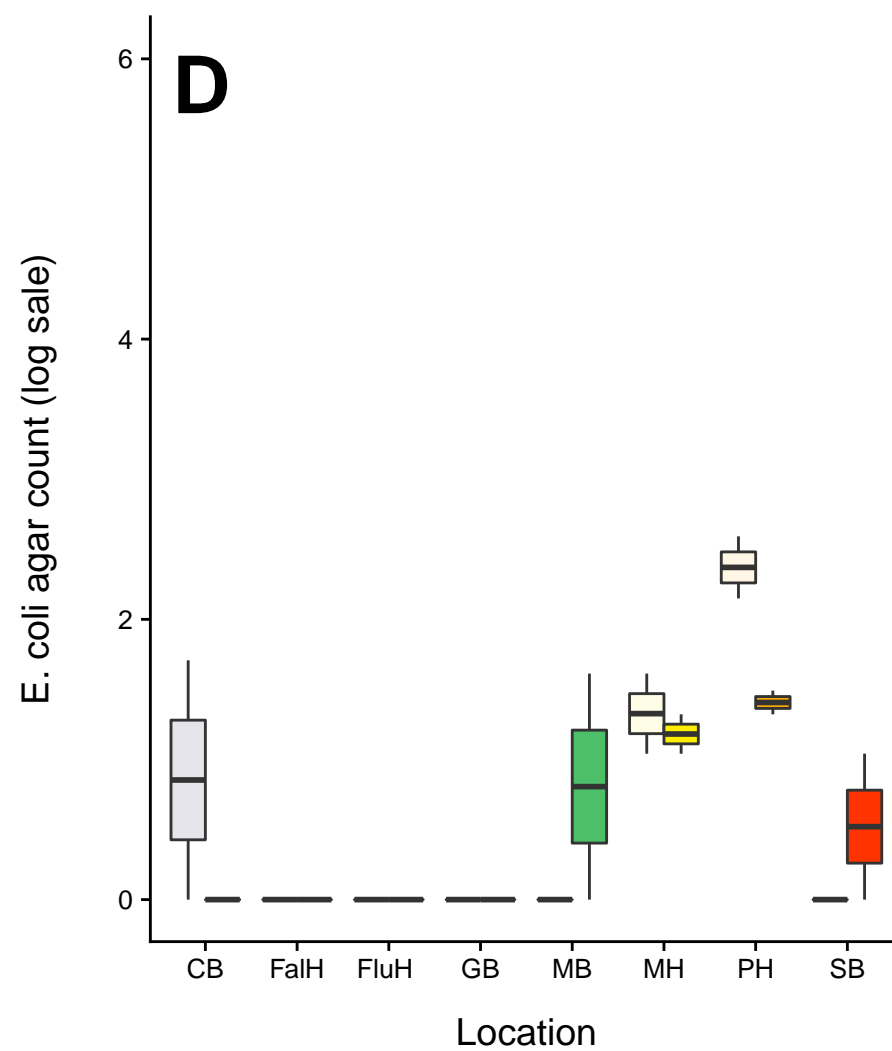

Supplement: Supplemental Information 11 — Boxplot summarizing variation in bacterial densities (expressed as log10 cells per 100 ml) across eight different locations and sample types (water versus sediment, where water samples are depicted first per location). Bacterial cell densities were enumerated using: (A) flow cytometry, (B) total LB agar counts, (C) total coliform agar counts and (D) E. coli and coliform counts (blue and pink colonies on coliform agar). Sample site abbreviations: CB: Castle Beach, FalH = Falmouth Harbour, FluH = Flushing Harbour, GB = Gyllyngvase Beach, MB = Maenporth Beach, MH = Mylor Harbour, PH = Penryn Harbour, SB = Swanpool Beach. [file peerj-07-6150-s011.pdf]

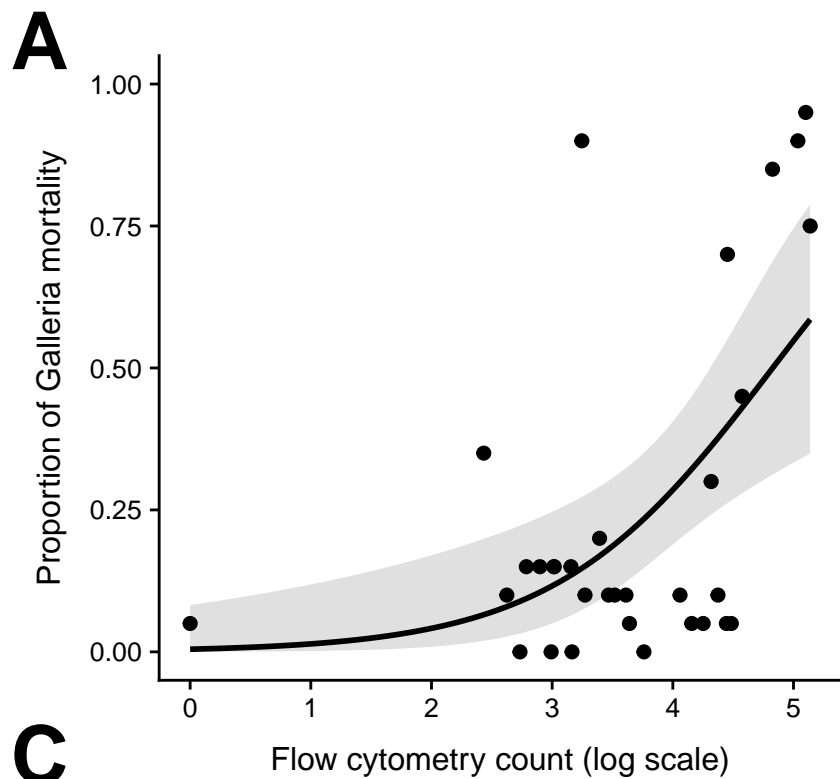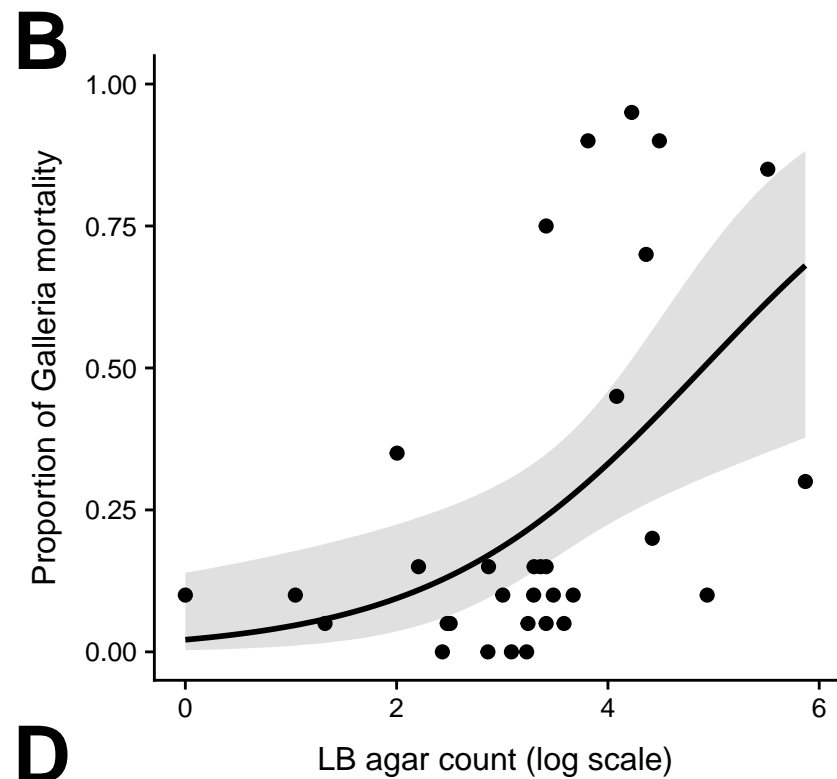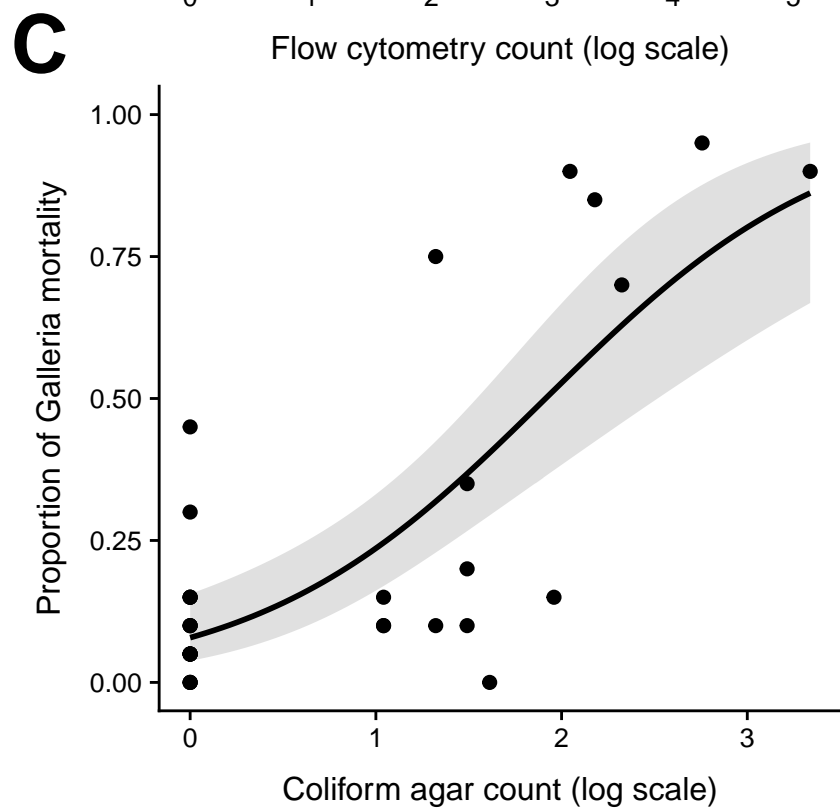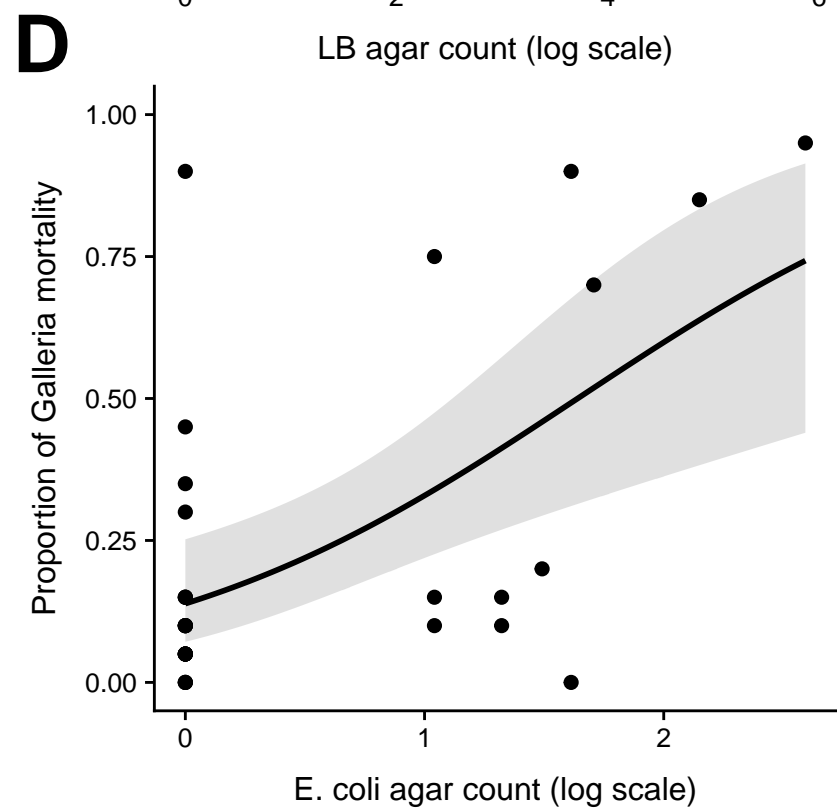

Supplement: Supplemental Information 12 — Plots depicting the relationship between Galleria mortality and bacterial cell density, estimated using: (A) flow cytometry, (B) total LB cell counts, (C) total coliform agar counts and (D) E. coli and coliform counts (blue and pink colonies on coliform agar). Lines and shaded area depict the fitted relationships ± standard error (see Table S1 for parameter estimates). [file peerj-07-6150-s012.pdf]
